# Impact of partial substitution of NaCl by KCl, and MgCl_2_ on physicochemical and sensory properties of cooked sausages during storage

**DOI:** 10.5713/ajas.18.0707

**Published:** 2019-01-02

**Authors:** Sang-Keun Jin, Sun-Jin Hur, Dong-Gyun Yim

**Affiliations:** 1 Department of Animal Resources Technology, Gyeongnam National University of Science and Technology, Jinju 52725, Korea; 2 Department of Animal Science and Technology, Chung-Ang University, Anseong 17546, Korea; 3 Department of Agricultural Biotechnology and Research Institute of Agriculture and Life Sciences, Seoul National University, Seoul 08826, Korea

**Keywords:** Sodium Substitution, NaCl, KCl, MgCl_2_, Meat Quality

## Abstract

**Objective:**

To determine the effect of three salting treatments (control, 100% NaCl; T1, 60% NaCl and 40% KCl; T2, 50% NaCl, 40% KCl, and 10% MgCl_2_) on meat quality of sausages during storage.

**Methods:**

Various types of curing salts were used for processing of salamis. Physico-chemical and sensory evaluation of the sausages were analyzed during 4 weeks of ripening.

**Results:**

The pH values and salinity contents were the highest in control, while they were the lowest in T2 at 4 weeks (p<0.05). Control and T1 had less thiobarbituric acid reactive substances than T2 at 2 weeks (p<0.05). The volatile basic nitrogen levels in T1 were similar to control and lower than T2 at 2 and 4 weeks (p<0.05). Moreover, T1 samples were redder and had a higher saturation index (C*) value when compared to the others. T2 showed greater hardness, gumminess and chewiness when compared with the control (p<0.05). Control was comparable to T1 for sensory attributes.

**Conclusion:**

Sodium chloride may be partially substituted with potassium chloride without negatively influencing meat quality of sausages up to 4 weeks of storage. These results could help to develop low-sodium sausages.

## INTRODUCTION

Sodium chloride (NaCl) acts as an essential ingredient in processed meat products because it improves quality characteristics such as texture, water-binding capacity, salty taste, flavor, and shelf-life [1]. Salt content is usually up to 2.0% to 4.0% for sausage mince [2]. However, excessive sodium intake may cause health issues leading to the development of hypertension and increase risks of cardiovascular diseases [3]. Thus, studies for low sodium sausages are vital to produce meat products with better health characteristics. Simply lowering NaCl without compromising sausage quality and safety is a big challenge [4]. Several studies have been performed to decrease the NaCl contents of processed meats by replacing NaCl with partial substitutes such as KCl, MgCl_2_, with similar features to NaCl [5–8]. KCl has the ‘Generally Recognized As Safe’ (GRAS) status by the U.S. Food and Drug Administration. The World Health Organization (WHO) recommended dosage of 3.5 g/d of potassium is found to be helpful in maintaining lower blood pressure levels [9]. NaCl and KCl have similar characteristics and potassium intake has not been linked to the development of hypertension and cardiovascular diseases [10,11]. However, high level (40%) of KCl can reduce the salty flavor and result in sensory changes linked to the development of a bitter and metallic flavor [12–15]. According to a previous study, it is possible to replace NaCl in sausages with KCl up to 30% to 40% [16]. Based on the results of our previous study (unpublished), we selected different concentrations of KCl and MgCl_2_, to evaluate the effect of partial substitution of sodium salts with these non-sodium salts. The aim of the present study was to investigate the effect of reduction of NaCl concentration to 60% or 50% and replacement with KCl and MgCl_2_ on the physico-chemical and sensory characteristics of cooked sausages during storage.

## MATERIALS AND METHODS

### Sausage preparation

As described in Table 1, salt-reduced pork sausages were control (100% NaCl), T1 (60% NaCl plus 40% KCl); or T2 (50% NaCl, 40% KCl plus 10% MgCl_2_). This formula was determined by preliminary experimental results with different replacement levels ranging from 10% to 60%. The amount of fat was adjusted to 12% without any fat replacer. Each of the groups composed of 3 different levels were manufactured in two batches (12 kg). The raw material (fresh pork ham and back fat) was ground to a particle size of 5 mm. The formulation of the batches included 72.44% lean pork, 11.2% back fat, 13.8% ice, 0.01% sodium nitrite, 0.2% sodium triphosphate, 0.5% sugar, 0.05% monosodium glutamate, and 0.4% spices. The whole components were mixed at a low speed for 15 min. Emulsification was achieved at a high speed (2,840 rpm) for approximately 8 min. The temperature of the mixture was maintained below 14°C. The mince was stuffed into 60 mm diameter regenerated fibrous casings. It was cooked in a smokehouse (Thematec Food Industry Co., Seongnam, Korea) until the internal temperature of the product ranged from 75°C to 78°C. After cooking, the sausages were cooled in an ice bath for 10 min, and vacuum packed immediately. The samples were stored under refrigerated conditions (3°C).

### Physico-chemical analysis

Sausage pH was estimated on 3 g of samples homogenized in distilled water (1:9) using a pH-meter (MP330, Mettler Toledo, Greifensee, Switzerland). The salinity of the sample was determined by the method of Chen [17]. After 2 and 4 weeks of storage, the samples were reweighed and the difference in weight was used to determine the purge loss. Thiobarbituric acid reactive substances (TBARS) values of sausages were measured by the method of Buege and Aust [18] and readings were taken using an X-MA 4000 spectrophotometer (Human Ltd., Seoul, Korea) at 530 nm. Volatile basic nitrogen (VBN) was determined as described by Conway [19]. Meat color of sausages from each formulation was measured with an appliance called Chroma Meter (CR-400, Minolta Co., Osaka, Japan). Based on CIE (Commission Internationale de l’Eclairage, International Commission on Illumination), the L* (lightness), a* (redness), and b* (yellowness) values of sausages were read. Chroma meter (a*^2^+b*^2^) and hue-angle (tan^−1^ [b*/a*]) were calculated from a* and b* values. To evaluate texture properties, a texture analyzer (TA-XT4, Stable Micro Systems, Godalming, UK) was used to evaluate samples with a 20 mm diameter and 10 mm thickness. Hardness, springiness, cohesiveness, gumminess, chewiness, and adhesiveness were obtained using the method of Bourne [20].

### Sensory evaluation

Sensory evaluation of sausages was performed by a sensory panel (12 trained panelists consisting of students and staff members from University) with training and experience on sensory evaluation. These panelists were trained for 4 weeks (2 times). Six among 12 panelists were randomly served per session and a total of 12 evaluation sessions were conducted. The samples were warmed, cut into cubes (5 cm^3^), and served to the panelists in a random order. Sensory evaluation was performed using a 9-point scale for the following criteria: color (pale to dark red); saltiness (not to very salty); bitterness (not to very bitter); chewiness (unacceptable to very acceptable); overall acceptability (unacceptable to very acceptable).

### Statistical analysis

All observations (3 treatments×3 batches×3 storage periods) were included in the statistical analysis. The experiment was carried out at various periods in the same location, and a randomized complete block design was utilized. Analysis of variance was conducted on all the variables analyzed using the general linear model procedure of SAS [21], which considers the additional level of target material or storage period as a fixed block effect and batch as a random term. In sensory variables, the model included the batch (treatment×storage) in addition to the main effects and their interaction, as well as the panelist in sensory evaluation, with the batch (treatment ×storage) used as the error term to determine the significance of the main effects and their interaction. The Duncan’s multiple range test was applied to evaluate differences among treatment means at the level of p<0.05.

## RESULTS AND DISCUSSION

The effect of sodium reduction or replacement on the physicochemical properties of sausage samples are presented in Table 2. The pH values were the highest in only NaCl formulation (control), while they were the lowest in T2 manufactured with a combination of KCl and MgCl_2_ (p<0.05). Gimeno [22] reported the influence of a mixture of NaCl, KCl, and MgCl_2_ on partial substitution of NaCl and observed a higher pH reduction in the lowered NaCl formulation when compared to only NaCl formulation. A similar trend was also described by Gimeno [23]. In our study, partial substitution of NaCl by KCl and MgCl_2_ significantly reduced the salinity content of sausages on 4 weeks of storage. Similar results were obtained by Campagnol [24] and Lorenzo [25]. Evidently, these modifications provide healthier sausage characteristics by reducing sodium intake. This alternative can reduce the incidence of hypertension and consequently the risk of cardiovascular diseases. Furthermore, the highest purge loss was observed in T1 samples after 2 and 4 weeks of storage (p<0.05). Pietrasik and Gaudette [26] reported that the samples formulated with 1.2% NaCl had higher purge loss after 4 and 8 weeks storage when compared to the samples formulated with 2% NaCl. TBARS values for 3 formulations did not differ at 0 and 4 weeks. The TBARS value in T1 sausages was similar to the control during storage. Similarly, Stanley [27] suggested no differences in TBARS values on partially replacing NaCl with KCl. This is also in accordance with the research by Andres [28], who determined that 6% NaCl had little pro-oxidant effect in ham, and the salt level added in this study (1.4%) was also below this range. Only control and T1 showed a lower TBARS values than T2 at 2 weeks. This result might be attributed to the higher amount of MgCl_2_ used in T2. Previous results were found by Lorenzo [25] and Zanardi [29], who showed that higher value of TBARS in sausage with the lowered NaCl formulation when compared to the NaCl formulation. On the contrary, Cheng [30] suggested that pork with 50% substitution of NaCl by KCl exhibited a lower value of TBARS during storage. The control, T1, and T2 groups maintained TBARS values lower than 0.5 mg of malondialdehyde/g over 4 weeks of storage which reflect the minimum rancidity level perceived by consumers [31]. In addition, the control had significantly lower VBN content than T1 and T2 at 0 week. The VBN content in T1 was similar to the control or lower than T2 at 2 and 4 weeks (p<0.05). VBN is used as a spoilage index because it can be increased by enzymatic degradation of protein as microorganisms grow in meat [32]. The results of our study showed that T1 samples containing NaCl 0.84% and KCl 0.56% had a positive effect on VBN in cooked sausages during storage. As expected, VBN value of sausages continuously increased during storage.

The effect of sodium reduction or replacement on color of sausage samples is shown in Table 3. T1 samples showed a higher L* value than control at 0 and 4 weeks (p<0.05). Similar results were shown by dos Santos [33]. T1 samples showed a higher a* value when compared to the other samples, whereas control samples showed a lower a* value at 0 and 2 weeks (p< 0.05). A similar result was shown in the research by Choi [34], in which a* values of samples with 60% NaCl and 40% KCl were higher than those of sausages with 100% NaCl. This agrees with the studies of Choi [34], who found that KCl sausages showed a redder color when compared with those produced using NaCl. Control samples showed a higher b* value when compared to the other samples at 0 and 4 weeks, whereas T2 samples showed a lower b* (p<0.05). Previous studies indicated no differences in color values when partially replacing NaCl with KCl or/and MgCl_2_ [25,35]. T1 samples showed a higher saturation index (C*) value when compared to the other samples, whereas T2 samples showed a lower C* regardless of storage period (p<0.05). Control samples showed the highest hue (*h*) (p<0.05).

As shown in Table 4, salt reduction had a detrimental effect on some of textural characteristics of sausages. Control samples showed lower hardness, gumminess, and chewiness values when compared to the other samples, whereas T2 with partial substitution of NaCl by the combination of KCl and MgCl_2_ showed a higher value (p<0.05). In addition, control showed a higher cohesiveness values when compared to the other samples (p<0.05). Laranjo [36] have reported that the higher sodium contents are, the higher the soluble protein extraction concentration, which contribute to a better binding of the meat mixture thereby improving cohesiveness. This is consistent with previous studies where partial replacement of NaCl by KCl and MgCl_2_ caused significant differences on the texture parameters in pork loin [37,38]. The higher hardness and chewiness values found for T2 could be related to reduction in the NaCl content [39]. As reported in the literature [24,39,40], the reduction or replacement of NaCl in sausages might change the texture.

The effect of sodium reduction or replacement on sensory test of sausage samples is shown in Table 5. Bitterness, juiciness, and chewiness were the three sensory attributes affected by the NaCl reduction. Samples with KCl received ratings closer to control for sensory scores. Color, saltiness (especially, considered crucial for low sodium sausages) and overall acceptability scores did not vary significantly among the treatments. This might be due to the fact that the panelists did not differentiate between low sodium samples (T1, T2) and control based on color and saltiness. Similarly, Guàrdia [6] did not observe significant differences in overall acceptability of samples formulated with 50% substitution of NaCl. Wu [41] found no adverse effects on sensory traits in meat by replacement of NaCl up to 40% with KCl. Moreover, for the attribute bitterness, the sensory panels could not distinguish between the control and treatments. Previous studies noted the detection of bitter taste in meat samples when NaCl was replaced by KCl above 40% [6]. The bitter/metallic flavor of KCl-incorporated low sodium products often leads to consumer rejection [3]. The panelists in our study, however, did not perceive any bitterness in KCl incorporated samples possibly because of the addition of spices and seasonings. According to the previous study, partial replacement of NaCl by KCl did not show significant differences on the sensory test in dry-cured hams when compared to the meat salted with 100% NaCl [42]. Additionally, the sensory quality of meat salted with 25% KCl, 15% CaCl_2_, and 5% MgCl_2_ did not show significant differences in relation to samples salted with 100% NaCl [43]. Collins [44] noted that use of 70% NaCl/30% KCl or 70% NaCl/30% MgCl_2_ mixtures did not lead to detectable differences with respect to flavor, tenderness, and overall acceptability when compared to the meat salted with 100% NaCl. These results are similar to those exhibited by Choi [34] who observed that the panelists could not distinguish a difference in color, saltiness, and overall acceptability. Armenteros [14] showed that samples salted with NaCl and KCl in equal amounts presented higher scores. T2 group had significantly lower values of juiciness than the others, whereas those exhibited higher values of chewiness at 4 weeks. Consequently, it was demonstrated that sensory traits of low sodium sausages were comparable to the control during storage of four weeks, indicating the possibility of replacing NaCl with up to 40% KCl in sausages without compromising the sensory quality.

## CONCLUSION

This study suggests that partially substituting NaCl with KCl could provide similar physicochemical and sensory properties when compared to conventional sausages with the addition of 100% sodium nitrite. Partial substitution of NaCl by KCl and MgCl_2_ reduced the pH values and salinity content of sausages. Sausages with 60% NaCl/40% KCl showed thiobarbituric acid, VBN content, and sensory attributes that were similar to the control samples. Unexpectedly, low sodium samples presented lower bitterness scores when compared to control at 4 weeks. In addition, the use of 40% KCl in sausages presented a redder color and had a higher saturation index (C*) value when compared to the other samples. Overall, the decrease of NaCl by 40% and its substitution by KCl in sausage manufacture can reduce the sodium content without detrimentally affecting physicochemical and sensory traits. However, further research should be performed to ensure the microbiological safety of food while substituting NaCl with KCl in meat products.

## Figures and Tables

**Table 1:** Percentages of sodium chloride, potassium chloride, and magnesium chloride used in the formulation of cooked sausage batter

Ingredient	Treatments1) (%)		

	Control	T1	T2
Pork lean meat	72.44	72.44	72.44
Pork backfat	11.2	11.2	11.2
Water (ice)	13.8	13.8	13.8
NaNO_2_	0.01	0.01	0.01
Phosphate	0.2	0.2	0.2
Sugar	0.5	0.5	0.5
MSG (monosodium glutamate)	0.05	0.05	0.05
Spices	0.4	0.4	0.4
NaCl (Sodium chloride)	1.4	0.84	0.7
KCl (Potassium chloride)	-	0.56	0.56
MgCl_2_ (Magnesium chloride)	-	-	0.14
Total	100.00	100.00	100.00

1) Control, 100% NaCl; T1, 60% NaCl and 40% KCl; T2, 50% NaCl, 40% KCl, and 10% MgCl_2_.

**Table 2:** Physico-chemical traits during storage of cooked sausages as influenced by the replacement of NaCl with KCl and/or MgCl_2_

Items	Treatments1)	Storage (weeks)			SEM	ANOVA2)

		0	2	4		
pH	C	6.66^A^^b^	6.89^A^^a^	6.16^A^^c^	0.075	T***3)
	T1	6.37^B^^b^	6.43^B^^a^	6.07^B^^c^	0.039	S***
	T2	6.35^B^^a^	6.31^C^^b^	6.03^C^^c^	0.035	T*S***
	SEM4)	0.03	0.06	0.01	-	
Salinity (%)	C	2.00^a^	2.00^a^	1.89^A^^b^	0.013	T***
	T1	2.00^a^	2.00^a^	1.22^B^^b^	0.090	S***
	T2	2.00^a^	2.00^a^	1.10^C^^b^	0.104	T*S***
	SEM4)	0.002	0.000	0.084	-	
Purge loss (%)	C	-	1.43^B^^a^	1.06^C^^b^	0.151	T***
	T1	-	1.68^A^^b^	2.54^A^^a^	0.258	S***
	T2	-	1.36^B^^a^	1.44^B^^a^	0.164	T*S***
	SEM4)		0.052	0.157	-	
TBARS (mg MA/kg)	C	0.38^a^	0.31^B^^b^	0.38^a^	0.010	S***
	T1	0.37^a^	0.32^B^^b^	0.39^a^	0.010	T*S*
	T2	0.39^a^	0.34^A^^b^	0.35^b^	0.009	
	SEM4)	0.006	0.006	0.010	-	
VBN (mg %)	C	7.52^B^^c^	8.43^B^^b^	9.15^B^^a^	0.16	T***
	T1	8.65^A^^b^	8.49^B^^b^	9.18^B^^a^	0.10	S***
	T2	8.42^A^^b^	8.77^A^^b^	13.24^A^^a^	0.54	T*S**
	SEM4)	0.13	0.06	0.47	-	

SEM, standard error of the mean; ANOVA, analysis of variance; TBARS, thiobarbituric acid reactive substances; VBN, volatile basic nitrogen.

1) C, NaCl 1.4%; T1, NaCl 0.84%, KCl 0.56%; T2, NaCl 0.7%, KCl 0.56%, MgCl_2_ 0.14%.

2) ANOVA, two-way ANOVA analysis among the treatments; T, treatment; S, storage.

3) ^*^p<0.05; ^**^ p<0.01; ^***^ p<0.001.

4) Standard error of mean (n = 16).

a–c Figures with different letters within a same row differ significantly (p<0.05).

A–C Figures with different letters within a same column differ significantly (p<0.05).

**Table 3:** Color characteristics during storage of cooked sausages as influenced by the replacement of NaCl with KCl and/or MgCl_2_

Items	Treatments1)	Storage (weeks)			SEM	ANOVA2)

		0	2	4		
L*	C	79.90^C^^c^	81.34^A^^a^	80.69^B^^b^	0.157	T***3)
	T1	80.79^A^^b^	80.42^B^^b^	81.45^A^^a^	0.125	S***
	T2	80.38^B^^b^	81.38^A^^a^	81.41^A^^a^	0.131	T*S***
	SEM4)	0.11	0.12	0.11	-	
a*	C	5.82^C^^b^	5.29^C^^c^	6.21^a^	0.097	T***
	T1	6.92^A^^a^	6.24^A^^c^	6.42^b^	0.076	S***
	T2	6.38^B^^a^	5.91^B^^b^	6.36^a^	0.073	T*S***
	SEM4)	0.12	0.10	0.04	-	
b*	C	8.57^A^^b^	8.74^B^^a^	8.57^A^^c^	0.097	T***
	T1	8.42^B^^b^	8.87^A^^a^	8.35^B^^b^	0.076	S***
	T2	7.86^C^^c^	8.24^C^^a^	8.08^C^^b^	0.073	T*S***
	SEM4)	0.08	0.07	0.05	-	
Chroma	C	10.37^B^^b^	10.21^B^^c^	10.59^A^^a^	0.041	T***
	T1	10.91^A^^a^	10.85^A^^a^	10.53^A^^b^	0.046	T*S***
	T2	10.14^C^	10.13^B^	10.28^B^	0.041	
	SEM4)	0.08	0.08	0.04	-	
Hue	C	55.81^A^^b^	58.81^A^^a^	54.09^A^^c^	0.504	T***
	T1	50.59^B^^c^	54.87^B^^a^	52.46^B^^b^	0.445	S***
	T2	51.01^B^^b^	54.34^B^^a^	51.81^C^^b^	0.404	T*S***
	SEM4)	0.63	0.49	0.26	-	

SEM, standard error of the mean; ANOVA, analysis of variance.

1) C, NaCl 1.4%; T1, NaCl 0.84%, KCl 0.56%; T2, NaCl 0.7%, KCl 0.56%, MgCl_2_ 0.14%.

2) ANOVA, two-way ANOVA analysis among the treatments; T, treatment; S, storage.

3) ^*^p<0.05; ^**^ p<0.01; ^***^ p<0.001.

4) Standard error of mean (n = 16).

a–c Figures with different letters within a same row differ significantly (p<0.05).

A–C Figures with different letters within a same column differ significantly (p<0.05).

**Table 4:** Texture profile during storage of cooked sausages as influenced by the replacement of NaCl with KCl and/or MgCl_2_

Items	Treatments1)	Storage (weeks)			SEM	ANOVA2)

		0	2	4		
Hardness (kg)	C	0.17^B^	0.16^B^	0.18^C^	0.004	T***3)
	T1	0.20^A^	0.18^AB^	0.20^B^	0.004	S***
	T2	0.19^A^^b^	0.19^A^^b^	0.21^A^^a^	0.003	
	SEM4)	0.004	0.004	0.004	-	
Surface hardness (kg)	C	0.17^B^	0.16^B^	0.18^B^	0.004	T***
	T1	0.20^A^	0.18^AB^	0.20^AB^	0.004	S***
	T2	0.20^A^^ab^	0.19^A^^b^	0.21^A^^a^	0.003	
	SEM4)	0.005	0.004	0.003	-	
Cohesiveness (%)	C	0.61^A^^a^	0.62^A^^a^	0.58^b^	0.007	T**
	T1	0.55^C^^b^	0.57^C^^b^	0.60^a^	0.006	T*S**
	T2	0.58^B^	0.59^B^	0.59	0.007	
	SEM4)	0.008	0.006	0.008	-	
Gumminess (kg)	C	0.11	0.10^B^	0.11^B^	0.002	T**
	T1	0.11^ab^	0.10^B^^b^	0.12^A^^a^	0.003	S***
	T2	0.11^b^	0.11^A^^b^	0.12^A^^a^	0.002	
	SEM4)	0.002	0.002	0.003	-	
Chewiness (kg, mm)	C	0.11	0.10^B^	0.11^B^	0.002	T**
	T1	0.11^a^	0.10^B^^b^	0.12^A^^a^	0.003	S***
	T2	0.11^b^	0.11^A^^b^	0.12^A^^a^	0.002	
	SEM4)	0.002	0.002	0.003	-	
Adhesiveness (kgf)	C	0.12^A^^a^	0.10^b^	0.11^ab^	0.003	S***
	T1	0.12^A^^a^	0.10^b^	0.11^a^	0.003	
	T2	0.10^B^	0.10	0.10	0.002	
	SEM4)	0.002	0.002	0.003	-	

SEM, standard error of the mean; ANOVA, analysis of variance.

1) C, NaCl 1.4%; T1, NaCl 0.84%, KCl 0.56%; T2, NaCl 0.7%, KCl 0.56%, MgCl_2_ 0.14%.

2) ANOVA, two-way ANOVA analysis among the treatments; T, treatment; S, storage.

3) ^*^p<0.05; ^**^ p<0.01; ^***^ p<0.001.

4) Standard error of mean (n = 16).

a–b Figures with different letters within a same row differ significantly (p<0.05).

A–C Figures with different letters within a same column differ significantly (p<0.05).

**Table 5:** Sensory profile during storage of cooked sausages as influenced by the replacement of NaCl with KCl and/or MgCl_2_

Items	Treatments1)	Storage (weeks)			SEM	ANOVA2)

		0	2	4		
Section color	C	7.08	7.17	6.58	0.114	
	T1	7.17	7.17	7.17	0.114	
	T2	7.08	7.00	7.17	0.093	
	SEM3)	0.050	0.131	0.124	-	
Saltiness	C	6.67^a^	6.75^a^	6.17^b^	0.169	S***4)
	T1	6.83	6.83	6.08	0.114	
	T2	6.83	6.42	6.50	0.143	
	SEM3)	0.135	0.107	0.09	-	
Bitterness	C	7.25^a^	6.92^a^	5.75^A^^b^	0.184	T**
	T1	7.00^a^	6.67^a^	4.67^B^^b^	0.276	S***
	T2	7.17^a^	6.75^a^	4.83^B^^b^	0.269	
	SEM3)	0.079	0.083	0.191	-	
Juiciness	C	6.50	5.83	6.00^A^	0.125	T*
	T1	6.08	5.67	5.50^A^	0.185	S*
	T2	6.33^a^	5.67^a^	4.83^B^^b^	0.188	T*S**
	SEM3)	0.129	0.116	0.16	-	
Chewiness	C	5.67^a^	4.67^B^^b^	4.92^B^^b^	0.168	T**
	T1	5.50^a^	5.92^A^^a^	4.67^B^^b^	0.190	T*S***
	T2	5.58	5.83^A^	5.83^A^	0.123	
	SEM3)	0.109	0.215	0.16	-	
Overall acceptability	C	6.33	6.67	6.08	0.105	
	T1	6.08	6.58	6.00	0.141	
	T2	6.50	6.45	6.38	0.119	
	SEM3)	0.115	0.140	0.144	-	

SEM, standard error of the mean; ANOVA, analysis of variance.

1) C, NaCl 1.4%; T1, NaCl 0.84%, KCl 0.56%; T2, NaCl 0.7%, KCl 0.56%, MgCl_2_ 0.14%.

2) ANOVA, two-way ANOVA analysis among the treatments; T, treatment; S, storage.

3) Standard error of mean (n = 16).

4) ^*^p<0.05; ^**^ p<0.01; ^***^ p<0.001.

a–b Figures with different letters within a same row differ significantly (p<0.05).

A–C Figures with different letters within a same column differ significantly (p<0.05).
